# Plasma tPA-Activity and Progression of Cerebral White Matter Hyperintensities in Lacunar Stroke Patients

**DOI:** 10.1371/journal.pone.0150740

**Published:** 2016-03-04

**Authors:** Ellen C. van Overbeek, Julie Staals, Iris L. H. Knottnerus, Hugo ten Cate, Robert J. van Oostenbrugge

**Affiliations:** 1 Department of Neurology, Maastricht University Medical Centre (MUMC), Maastricht, the Netherlands; 2 Cardiovascular Research Institute Maastricht, MUMC, Maastricht, the Netherlands; 3 Department of Neurology, Medisch Spectrum Twente, Enschede, the Netherlands; 4 Department of Internal Medicine, MUMC, Maastricht, the Netherlands; University of Rouen, France, FRANCE

## Abstract

**Introduction:**

Tissue plasminogen activator (tPA)-activity and plasminogen activator inhibitor type 1 (PAI-1) antigen are considered to be haemostasis-related markers of endothelial activation and relate to presence of cerebral white matter hyperintensities (WMH) as was earlier shown in a cross-sectional study. We investigated whether tPA-activity and PAI-1 levels are associated with WMH progression in a longitudinal study.

**Methods:**

In 127 first-ever lacunar stroke patients in whom baseline brain MRI and plasma levels of tPA-activity and PAI-1-antigen were available, we obtained a 2-year follow-up MRI. We assessed WMH progression by a visual WMH change scale. We determined the relationship between levels of tPA-activity and PAI-1 and WMH progression, by logistic regression analysis.

**Results:**

Plasma tPA-activity was associated with periventricular WMH progression (OR 2.36, 95% CI 1.01–5.49, with correction for age and sex and baseline presence of WMH), but not with deep or any (periventricular and/or deep) WMH progression. PAI-1 levels were lower in patients with WMH progression, but these results were not significant.

**Conclusion:**

We found a relationship between plasma tPA-activity and progression of periventricular WMH. More research is needed to determine whether there is a (direct) role of tPA in the development and progression of WMH.

## Introduction

Tissue plasminogen activator (tPA) and plasminogen activator inhibitor type 1 (PAI-1) antigen are components of the fibrinolytic system. It is hypothesized that tPA and PAI-1 are haemostasis-related markers of endothelial function [1–4]. Endothelial dysfunction, probably systemic and not just in the cerebral vessels, is considered one of the main underlying initiating causes of cerebral small vessel disease (cSVD) [5]. One of the most prevalent manifestations of cSVD are white matter hyperintensities (WMH) visible on brain MRI [5]. WMH are associated with stroke risk, mortality, gait abnormalities and cognitive impairment [6]. A better understanding of the pathogenesis and associated factors of WMH may help developing future treatment strategies.

Earlier, we showed that high levels of plasma tPA-activity and low levels of PAI-1 were cross-sectionally associated with extensive WMH in lacunar stroke patients [7]. The aim of this study was to determine whether plasma tPA-activity and PAI-1 levels are associated with WMH progression.

## Methods

### Ethics statement

The study was approved by the medical ethics committee of the University Hospital Maastricht and Maastricht University (METC azM / UM). All patients gave written informed consent.

### Patients

At baseline 149 first-ever lacunar stroke patients were included consecutively from a prospective ongoing lacunar stroke project as described before [7]. Brain MRI and tPA-activity and PAI-1-antigen measurements were obtained at baseline. Patients with extensive vascular comorbidity, vasculitis and systemic lupus erythematosus, which can influence levels of tPA-activity and PAI-1, were excluded. Patients were offered a clinical brain MRI after 2 years of follow-up.

### MRI acquisition

MR images were obtained with a1.5 Tesla (T) or 3T MRI scanner (Philips). For this study standard axial T2-weighted fast spin echo and fluid attenuated inversion recovery sequences were used. Scanning parameters were unchanged between baseline and follow-up.

Baseline MR images were obtained at a median of 30 days (interquartile range (IQR) 8–76 days) after the stroke event. Follow-up MR images were performed 2 years after the baseline imaging (median 25, IQR 24–26 months).

### MRI scoring

#### Baseline

The presence of WMH at baseline was assessed using Fazekas scale. Presence of extensive white matter hyperintensities (WMH) was defined as T2-weighted (early) confluent deep hyperintensities (dWMH) (Fazekas 2 and 3) or irregular periventricular hyperintensities (pWMH) extending into the deep white matter (Fazekas 3) [8]. These Fazekas scores are histopathologically related cSVD [9].

#### Follow-up

The WMH change scale as proposed by Prins et al. was used to assess progression of WMH on follow-up MRI [10]. This scale enables to score white matter changes in three periventricular regions (frontal caps, lateral bands and occipital caps) and four deep regions (frontal, parietal, temporal, occipital). Increase of WMH in a region (+1) was defined as new lesions or increase of existing lesion. This adds up to a progression score ranging from -3 to +3 in periventricular regions and -4 to +4 in deep regions. WMH progression in the study was defined as a score ≥ 1. Two experienced vascular neurologists, who were blinded for baseline levels of tPA-activity and PAI-1, independently assessed WMH progression. In case of disagreement a consensus meeting was held. The inter-rater agreement, expressed by Cohen’s kappa (ĸ), was substantial for total progression at follow-up (0.62).

### tPA-activity and PAI-1

#### Samples

At baseline, but at least 3 months after the acute ischemic event, fasting blood samples were drawn in all patients by venepuncture of a vein in the antecubital fossa. Because the levels of tPA-activity and PAI-1 are characterized by diurnal variation, samples were drawn between 8:30 a.m. and 10:00 a.m. in all patients. Prescribed medication including antiplatelet agents and statins were continued at time of blood withdrawal. The first tube was discarded to minimize puncture-related coagulation activation. Consecutively the blood was divided over a tube containing citrate anticoagulant at low pH (to prevent formation of tPA–PAI-1 complex and preserving components of the fibrinolytic system; Stabilyte tube; Biopool) and a tube containing 3.2% sodium citrate. Platelet-poor plasma was prepared by a 2-step centrifugation process (5 minutes at 2000 *g* and 10 minutes at 11 000 *g* at room temperature). Plasma aliquots were stored at -80°C and defrosted at 37°C before analysis.

#### Laboratory assays

A chromolize tPA kit (Biopool) was used to determine tPA-activity. PAI-1 antigen levels were measured by TECHNOZYM PAI-1 antigen enzyme-linked immunosorbant assay reagent kit by Technoclone (commercially available). The coefficients of variation for tPA-activity and PAI-1 was <10% and 3% to 10%, respectively.

### Statistical analysis

Statistical analyses were performed using IBM SPSS Statistics 21. Differences between groups were determined using χ^2^-test, *t* test, or Mann–Whitney test, where appropriate. Categorical variables are presented as frequencies (%), normally distributed data as mean ± SD and variables with skewed distributions are presented as median and interquartile ranges.

We assessed the relationship between plasma levels of tPA-activity and levels of PAI-1 (independent variables) and WMH progression (dependent variable) by binary logistic regression analysis adjusted for age and sex. We performed three separate analyses: pWMH progression, dWMH progression and progression of WMH in any (periventricular and/or deep) region respectively. In additional exploratory analyses we adjusted for cardiovascular risk factors (hypertension, diabetes mellitus, hypercholesterolemia and current smoking), 24-hour mean arterial pressure (MAP) at follow-up and presence of extensive deep or periventricular WMH at baseline by adding them one at a time to the model.

Odds ratios (OR) are given with 95% confidence interval (CI). Statistical significance was considered at *P* < 0.05.

## Results

### Patients

Of 149 included first-ever lacunar stroke patients at baseline, follow-up MRI at 2 years (mean 25.2 ± 2.2 months) was obtained in 132 patients. Two patients died of non-neurological cause and 15 patients had no follow-up MRI. Another 5 patients were excluded because of inadequate scan data, leaving 127 (85.2%) patients in our analyses. Baseline characteristics of these patients are presented in Table 1. Any (periventricular and/or deep) WMH progression was present in 61 (48%) patients. Patients with WMH progression were significantly older and had more extensive WMH at baseline. Thirthy-three (26%) patients had pWMH progression and 50 (39%) had dWMH progression.

**Table 1:** Baseline characteristics.

	All patientsN = 127	No WMH progression n = 66	Any WMH Progression n = 61	No pWMH progression n = 94	pWMH progression n = 33	No dWMH progression n = 77	dWMH progression n = 50
Male	78 (61.4)	43 (65.2)	35 (57.4)	14 (62.8)	19 (57.6)	50 (64.9)	28 (56.0)
Age (years)	62.5 ± 11.7	58.6 ± 11.6	66.7 ± 10.3 ^a^	60.1 ± 11.8	69.2 ± 8.3 ^a^	60.0 ± 11.7	66.3 ± 10.7 ^a^
tPA-activity (IU/mL)	0.65 (0.36–1.11)	0.57 (0.35–0.97)	0.72 (0.37–1.34)	0.57 (0.34–0.97)	1.05 (0.48–1.54) ^a^	0.60 (0.36–1.06)	0.71 (0.36–1.44)
PAI-1 (ng/mL)	34.1 (17.6–69.5)	37.9 (18.1–74.0)	27.1 (15.8–57.3)	37.4 (19.2–78.6)	23.0 (13.1–42.2)	35.4 (19.7–69.7)	26.6 (12.0–69.8)
Extensive periventricular WMH at baseline (Fazekas 3)	34 (26.8)	7 (10.6)	27 (44.3) ^a^	15 (16.0)	19 (57.6) ^a^	13 (16.9)	21 (42.0) ^a^
Extensive deep WMH at baseline (Fazekas 2+3)	37 (29.1)	8 (12.1)	29 (47.5) ^a^	20 (21.3)	17 (51.5) ^a^	9 (11.7)	28 (56.0) ^a^
Hypertension	85 (66.9)	42 (63.6)	43 (70.5)	63 (67.0)	22 (66.7)	49 (63.6)	36 (72.0)
Diabetes Mellitus	17 (13.4)	6 (9.1)	11 (18.0)	12 (12.8)	5 (15.2)	8 (10.4)	9 (18.0)
Hypercholestrolemia	96 (75.6)	48 (72.7)	48 (78.7)	68 (72.3)	28 (84.8)	57 (74.0)	39 (78.0)
Current smoking	54 (42.5)	29 (43.9)	25 (41.0)	39 (41.5)	15 (45.5)	34 (44.2)	20 (40.0)
24-hour mean arterial pressure (mmHg) at follow-up^b^	100.9 ± 9.7	100.8 ± 10.3	101.0 ± 9.1	100.0 ± 9.7	103.1 ± 9.3	101.5 ± 10.0	100.0 ± 9.2

Data are presented as mean ± SD, median (interquartile range) or number (%). WMH: white matter hyperintensities. any WMH progression: progression of periventricular and/or deep WMH.

^a^ p < 0.01 (progression vs. no progression)

^b^ 7 missing data

### WMH progression and tPA-activity and PAI-1

Plasma tPA-activity was higher in patients with pWMH progression compared to those without (Table 1). In logistic regression analyses with correction for age and sex, tPA-activity was associated with pWMH progression (OR 2.55, 95% CI 1.14–5.71, Table 2). Correction for age, sex and 24-hour MAP also gave a significant association between tPA-activity and pWMH progression (OR 2.34, 95% CI 1.03–5.34), and the results were similar with correction for sex, age and any of the cardiovascular risk factors (including hypertension), as well as with correction for age, sex and the presence of extensive pWMH at baseline (OR 2.36, 95% CI 1.01–5.49). Plasma tPA-activity was also associated to any (periventricular and/or deep) WMH progression and dWMH progression. However, in regression analyses with adjustment for sex and age there was no significant association between plasma tPA-activity and dWMH or any (periventricular and/or deep) WMH progression. PAI-1 levels were (not significantly) lower in patients with WMH progression compared to those without progression (Table 1). However there was no significant association between PAI-1 levels and WMH progression in regression analysis (Table 2).

**Table 2:** Binary logistic regression analysis with progression of white matter hyperintensities as dependent variable.

	Any WMH progression		Periventricular WMH progression		Deep WMH progression	
	Unadjusted	Adjusted for sex and age	Unadjusted	Adjusted for sex and age	Unadjusted	Adjusted for sex and age
tPA- activity (IU/mL)	2.38 (1.18–4.81)^a^	1.89 (0.89–4.02)	3.10 (1.47–6.54)^a^	2.55 (1.14–5.71)^a^	2.16 (1.09–4.29)^a^	1.77 (0.86–3.65)
PAI-1 (ng/mL)	1.00 (0.99–1.00)	1.00 (0.99–1.00)	0.99 (0.98–1.00)	1.00 (0.98–1.00)	1.00 (0.99–1.00)	1.00 (0.99–1.01)

All results are presented as OR (95%CI). WMH: White matter hyperintensities. Any WMH progression: periventricular and/or deep WMH progression.

^a^ p < 0.05

## Discussion

We found that higher baseline plasma tPA-activity was associated with progression of pWMH after 2 years of follow-up in lacunar stroke patients. Our results are in concordance with our previous cross-sectional findings [7]. Until now, longitudinal studies on the relation between tPA and WMH were lacking.

Endothelial cells are a source of plasma tPA [11]. It has been proposed that increased levels of plasma tPA-activity may reflect increased endothelial activation state, or endothelial dysfunction [1–4]. Endothelial dysfunction is thought to be an important initiating cause of cSVD, resulting in WMH [5]. Moreover, endothelial dysfunction in cSVD is considered not to be an isolated process in the brain, but a general systemic microvascular dysfunction [12]. SVD in the brain has been associated with SVD in other organs such as kidneys and retinal vessels [12]. cSVD has also been associated with other plasma markers for endothelial dysfunction [1, 13].

Although hypothesized before by others that tPA-activity might be regarded as a marker of (systemic) endothelial activation linked to cSVD, our study cannot show whether the association between progression of WMH and plasma tPA-activity is the result of endothelial activation and one can only speculate about it. Other factors besides endothelial dysfunction may also influence plasma tPA-activity. These include inflammation, seasonal variation, tPA gene polymorphisms and others [11]. These factors could also be involved in the link between tPA and cSVD.

Reflecting on the association between tPA and cSVD, we might also consider the local effect: tPA is locally expressed within the brain and release of tPA in the neurovascular unit may induce increased permeability of the blood-brain barrier [14–16]. This may promote leakage of plasma components into the perivascular space and damage of the white matter [5, 17]. However, it is unclear whether plasma levels of tPA-activity reflect local concentrations within the brain parenchyma and the microvasculature of the brain. Also, the effects of tPA in the brain are multiple [14, 18] and there are also data that suggest that neuronal released tPA has a neuroprotective role [14]. In animal studies, it was shown that tPA expression in the white matter is decreased with aging and this was accompanied by an increased vulnerability of the white matter to ischemic damage [19]. Furthermore, as once said, we only found an association between plasma tPA-activity and progression of pWMH and cannot conclude anything on cause-effect relationships.

Few other studies reported on the association between tPA and cSVD. We found earlier that plasma tPA-activity was higher in lacunar stroke patients with WMH compared to patients without WMH [7]. Also, plasma tPA was found to be higher in lacunar (small vessel) stroke patients compared to non-stroke individuals [1]. A tPA gene polymorphism has been associated with lacunar stroke subtype [20]. Higher levels of plasma tPA have also been found in other cardiovascular diseases or risk factors, such as in diabetes [21], myocardial infarction [22] and familial hyperlipidemia [23].

Next to the association between pWMH and plasma tPA-activity, we also found an association between plasma tPA-activity and dWMH, however these results lost significance after correction for sex and age. Perhaps the effect is small and a larger population is needed to confirm an association. On the other hand, several studies report different neuropathological findings, risk factors and rates of progression between dWMH and pWMH, which could indicate differences in underlying pathophysiology [24]. Also, presence or progression of pWMH, but not dWMH, has been related to cognitive decline [24].

Although we found plasma PAI-1 levels to be lower in patient with progression of WMH, these results were not significant. PAI-1inhibits tPA-activity and it is also hypothesized that PAI-1 may play a role in maintaining the blood-brain barrier by directly enhancing endothelial tight junction properties [25, 26].

Our study has some limitations. Plasma tPA-activity and PAI-1-antigen levels were only measured once at baseline. However, a previous study found that the plasma levels of tPA and PAI-1 did not significantly change over time in stroke patients [27]. Furthermore, blood was drawn after the acute stroke phase as we were not aiming to measure results of acute ischemic injury. Secondly, MR imaging was performed on a 1.5T or a 3T scanner. In most patients (106, 83.5%), baseline and follow-up scans were performed on the same type of scanner. In 21 (16.5%) patients a 3T baseline and 1.5T follow-up scan was done, which could have led to underestimation of WMH progression. Finally, we did not use volumetric WMH measurements, nonetheless we used a visual WMH change scale known to be highly correlated to changes in WMH volume [10, 28].

## Conclusions

We found a relationship between plasma tPA-activity and pWMH progression in first-ever lacunar stroke patients. More research is needed to determine whether there is a (direct) role of tPA in the development and progression of WMH.

## Supporting Information

S1 Dataset vOverbeek tPA and progression of WMH. (PDF)
