# Supplementary material for: Plasma tPA-Activity and Progression of Cerebral White Matter Hyperintensities in Lacunar Stroke Patients
Source: PLoS One. 2016 Mar 4;11(3):e0150740. doi: 10.1371/journal.pone.0150740 (PMC4778794; doi:10.1371/journal.pone.0150740)
Supplement: S1 Dataset — (PDF) [file pone.0150740.s001.pdf]

registration number	age	sex	tPA-activity	PAI-1	baseline pWMH	baseline dWMH	progression WMH	progression pWMH	progression dWMH	hypertension	DM	smoking	hypercholestroleamia	24h MAP at follow-up
1	67	1	0.37	121.49	1	1	0	0	0	1	0	0	1	97.19
2	47	0	1.07	22.67	0	0	0	0	0	1	0	1	0	92.85
3	71	0	0.29	34.35	0	0	0	0	0	0	1	1	1	93.33
4	56	0	1.44	9.33	0	0	0	0	0	0	0	1	1	92.59
5	47	1	0.31	41.75	0	0	0	0	0	1	1	1	0	140.19
6	68	1	1.58	31.52	0	0	0	0	0	1	0	0	1	93.69
7	42	0	0.27	59.78	0	0	0	0	0	0	0	1	1	106.46
8	50	1	1.11	11.35	0	0	0	0	0	0	0	1	1	84.76
9	46	1	0.6	33.46	0	0	0	0	0	1	0	0	0	119.15
10	65	0	1.06	15.92	1	0	0	0	0	0	0	1	1	115.32
11	67	0	0.72	34.95	1	0	1	1	0	0	0	0	1	94.64
12	61	1	1.38	15.72	0	0	0	0	0	0	0	0	0	93.93
13	63	1	0.33	27.12	0	0	1	0	1	1	0	0	1	104.23
14	54	1	0.33	69.96	0	0	0	0	0	1	0	1	1	102.43
15	57	1	0.72	69.54	0	0	1	0	1	1	0	1	1	104.99
16	79	0	0.22	39.2	1	1	1	0	1	1	0	0	1	100.49
17	83	1	0.76	41.37	0	0	1	1	0	0	0	0	0	101.32
18	85	1	0.62	78.59	0	0	1	0	1	0	0	1	1	94.17
19	51	1	0.49	72.46	0	0	0	0	0	0	0	0	1	109.99
20	61	1	0.24	100.8	0	0	1	0	1	1	1	1	0	97.35
21	46	0	0.34	123.62	0	0	0	0	0	0	0	0	1	98.65
22	64	1	0.43	140.52	0	0	0	0	0	1	0	1	1	102.29
23	64	1	1.06	23.3	0	0	1	1	0	1	1	0	1	112.19
24	60	1	0.29	179.78	0	0	1	0	1	1	0	1	1	103.9
25	67	0	1.06	79.43	1	1	1	1	1	1	0	1	1	
26	67	0	0.31	121.49	0	1	1	0	1	1	0	0	1	
27	77	0	1.71	18.71	1	1	1	1	1	0	0	0	1	90.03
28	81	1	1.71	6.33	1	1	1	1	1	0	0	1	1	91.33
29	68	0	1.26	33.12	0	0	1	1	1	1	0	1	1	102.45
30	76	1	1.33	12.36	0	0	0	0	0	1	0	1	0	100.68
31	74	1	1.24	33.23	1	1	0	0	0	0	0	0	1	91.79
32	52	1	0.71	29.59	0	0	0	0	0	1	0	1	0	125.51
33	68	1	0.29	17.64	0	0	1	1	1	1	0	0	0	112.75
34	60	0	0.12	168.52	0	0	1	1	0	1	0	0	1	90.34
35	66	1	1.8	11.04	1	1	1	1	1	1	1	0	1	91.65
36	68	1	0.94	8.98	0	0	0	0	0	1	0	0	0	129.42
37	73	0	0.36	83.09	0	1	1	0	1	0	0	0	1	96.07
38	63	0	0.58	53.84	0	0	0	0	0	1	0	1	0	97.33
39	74	1	0.39	70.6	1	1	1	1	1	0	0	0	1	102.02
40	54	1	0.83	120.42	0	0	0	0	0	0	0	1	1	91.29
41	77	1	0.18	20.46	1	1	1	1	1	1	0	0	1	94.13
42	64	1	1.04	5.06	1	1	1	0	1	1	0	1	1	104.71
43	64	1	0.13	195.02	0	0	0	0	0	0	0	1	0	99.02
44	77	0	0.13	229.2	0	0	0	0	0	1	0	0	1	93.51
45	51	1	1.99	5.49	0	0	1	0	1	1	1	0	1	108.53
46	52	1	0.62	58.15	0	1	1	0	1	0	0	1	0	98.84
47	72	1	0.68	17.02	0	0	0	0	0	0	0	0	1	105.6
48	76	1	0.27	27.89	1	1	0	0	0	1	0	1	1	88.72
49	63	1	0.49	86.9	0	1	0	0	0	1	0	1	1	112.13
50	77	1	0.32	44.45	0	0	0	0	0	1	1	1	1	103.41
51	79	0	1.68	19.99	1	1	1	1	0	1	0	0	1	100.65
52	79	1	0.84	14.81	1	1	0	0	0	1	0	1	0	100.5
53	75	0	1.25	38.78	0	0	0	0	0	0	0	1	1	103.47
54	71	1	1.17	10.16	1	0	1	1	0	1	1	1	1	106.43

55	79	1	0.48	20.51	1	1	1	1	1	1	1	0	1	110.46
56	81	0	2.1	11.92	0	0	1	0	1	1	0	0	0	75.4
57	53	0	2.1	7.67	0	0	1	1	1	0	0	1	1	114.34
58	80	1	0.79	37.93	1	1	1	1	1	1	1	0	0	118.5
59	68	0	0.35	17.55	0	1	1	1	1	0	0	0	1	108.21
60	79	0	0.72	22.36	0	0	1	1	0	0	0	1	1	107.69
61	70	1	0.48	42.94	1	1	1	1	1	1	0	1	1	98.25
62	74	1	2.05	19.93	1	1	1	0	1	1	0	0	1	111.11
63	69	0	0.49	34.55	0	0	1	0	1	1	1	0	1	96.32
64	77	1	0.69	19.47	0	0	0	0	0	1	0	1	1	110.23
65	40	0	0.24	84.35	0	0	0	0	0	1	0	0	1	
66	50	0	1.33	10.09	1	1	0	0	0	0	0	1	1	91.75
67	67	0	1.85	19.9	0	0	0	0	0	1	0	0	0	100.45
68	52	1	0.14	117.21	0	0	0	0	0	1	0	1	1	90.43
69	59	1	0.39	35.36	0	0	0	0	0	1	0	0	1	92.06
70	47	0	0.43	78.7	0	0	0	0	0	1	0	0	1	94.86
71	78	0	2.06	9.59	1	1	1	1	1	1	0	0	1	107.7
72	56	1	0.69	56.49	0	0	1	1	0	1	0	1	0	111.62
73	45	1	0.58	39.47	0	0	0	0	0	0	0	1	1	
74	60	0	0.24	96.58	1	0	1	0	1	1	0	1	1	84.58
75	47	0	0.77	37.1	0	0	0	0	0	1	0	0	1	90.65
76	65	0	1.41	22.99	0	1	1	1	1	1	0	0	1	102.47
77	65	1	2.1	34.07	0	1	1	1	1	1	0	0	1	108.25
78	50	1	0.31	69.54	0	0	0	0	0	1	0	1	1	102.43
79	63	1	0.74	21.59	0	0	1	0	1	0	0	0	0	96.76
80	71	1	1.62	10.4	1	1	1	0	1	1	1	0	0	102.44
81	65	0	0.62	60.03	0	0	0	0	0	1	0	0	1	
82	60	0	0.4	20.51	0	0	1	0	1	1	1	0	1	86
83	56	0	0.73	79.65	0	0	0	0	0	0	0	1	0	100.13
84	67	0	0.48	139.47	0	0	0	0	0	1	0	0	1	98.52
85	60	1	0.57	43.45	0	0	0	0	0	1	1	0	1	105.91
86	46	0	0.3	99.87	0	0	1	0	1	1	0	0	0	106.45
87	40	1	0.29	156.15	0	0	0	0	0	1	0	0	1	104.2
88	61	1	0.82	14.13	1	0	1	1	1	1	0	1	1	93.51
89	61	1	0.41	38.99	0	0	0	0	0	1	0	0	1	104.27
90	49	0	1.33	11.52	0	1	1	1	1	0	0	1	0	108.86
91	77	0	0.56	29.93	1	1	1	0	1	1	0	0	0	99.04
92	74	0	2.01	6.68	1	1	1	1	1	0	0	0	1	90.11
93	65	0	0.81	12.01	1	1	1	1	1	1	0	1	1	122.46
94	62	1	0.31	49.6	1	0	1	1	0	1	0	1	1	115.72
95	55	1	0.36	43.64	0	0	0	0	0	0	0	0	0	90.16
96	42	1	1.07	6.53	0	0	0	0	0	0	1	1	1	106.52
97	73	0	0.58	66.06	0	0	0	0	0	1	1	0	1	
98	45	1	0.38	43.69	0	0	0	0	0	1	0	0	0	99.12
99	74	1	1.11	71.85	0	0	1	1	1	1	0	1	1	96.73
100	79	1	1.17	9.68	0	0	0	0	0	0	0	0	0	104.74
101	40	1	0.26	33.05	0	0	0	0	0	0	0	0	1	102.18
102	70	1	1.06	7.19	0	0	0	0	0	1	0	0	1	108.33
103	80	0	0.7	10.42	1	1	1	0	1	1	0	0	1	93.26
104	65	1	0.86	22.87	0	0	1	0	1	1	0	0	1	104.23
105	36	0	1.7	94.37	0	0	0	0	0	1	0	1	1	102.64
106	64	1	1.23	27.5	1	0	1	1	0	1	0	0	1	108.56
107	59	1	0.54	18.42	0	0	0	0	0	0	0	0	1	102.65
108	67	1	1.05	26.25	1	0	1	1	0	0	0	1	1	107.43
109	60	0	0.37	37.71	1	1	1	0	1	0	0	1	0	93.15

110	52	1	0.38	83.22	0	0	0	0	0	0	0	1	1	100.3
111	54	1	1.13	30.43	0	0	0	0	0	1	0	0	1	102.41
112	68	1	0.52	20.7	1	1	0	0	0	1	0	0	1	98.73
113	65	1	1.37	112.91	0	0	1	0	1	0	0	1	1	102.91
114	49	1	0.23	6.31	0	0	0	0	0	1	0	0	0	108.36
115	66	1	0.41	126.81	0	0	0	0	0	1	0	0	0	91.4
116	49	1	0.45	42.88	0	0	1	0	1	1	0	1	1	
117	47	0	0.37	34.95	0	0	0	0	0	1	0	0	1	94.57
118	69	0	0.67	44.79	0	0	0	0	0	1	0	0	1	94.73
119	45	0	0.36	32.77	0	0	0	0	0	1	0	0	1	102.03
120	63	1	0.88	6.3	0	0	0	0	0	0	0	0	0	82.95
121	53	0	1.53	6.3	0	1	1	0	1	1	0	0	1	99.75
122	53	1	0.34	53.96	0	0	0	0	0	0	0	1	1	110.12
123	65	1	0.68	45.99	0	1	1	0	1	1	1	0	1	89.3
124	73	1	0.28	163.38	0	0	1	1	1	1	0	1	1	88.48
125	33	1	0.03	26.05	0	0	1	0	1	0	0	0	1	93.86
126	57	1	0.82	6.3	0	1	0	0	0	1	0	0	1	90.64
127	55	0	0.65	6.3	0	0	0	0	0	1	0	0	1	88.67

sex: 0 = female
1= male

other: 0 = no
1 = yes
